# SMO-M2 mutation does not support cell-autonomous Hedgehog activity in cerebellar granule cell precursors

**DOI:** 10.1038/s41598-019-56057-y

**Published:** 2019-12-23

**Authors:** Marialaura Petroni, Maria Sahùn Roncero, Valentina Ramponi, Francesca Fabretti, Vittoria Nicolis Di Robilant, Marta Moretti, Vincenzo Alfano, Alessandro Corsi, Simone De Panfilis, Maria Giubettini, Stefano Di Giulio, Carlo Capalbo, Francesca Belardinilli, Anna Coppa, Francesca Sardina, Valeria Colicchia, Flaminia Pedretti, Paola Infante, Beatrice Cardinali, Alessandra Tessitore, Gianluca Canettieri, Enrico De Smaele, Giuseppe Giannini

**Affiliations:** 1 Center for Life Nano Science@Sapienza, Istituto Italiano di Tecnologia, 00161 Rome, Italy; 2 Department of Molecular Medicine, University La Sapienza, 00161 Rome, Italy; 3 Department Biology and Biotechnology Charles Darwin, University La Sapienza, 00161 Rome, Italy; 4 CrestOptics Spa, 00165 Rome, Italy; 5 Department Experimental Medicine, University La Sapienza, 00161 Rome, Italy; 6 Institute of Cell Biology and Neurobiology, National Research Council, Campus A. Buzzati-Traverso, Monterotondo, 00015 Rome, Italy; 7 Department of Biotechnological and Applied Clinical Sciences, University of L’Aquila, 67100 L’Aquila, Italy; 8 Istituto Pasteur-Fondazione Cenci Bolognetti, 00161 Rome, Italy; 9 Present Address: Epigenetics and epigenomic of hepatocellular carcinoma Cancer Research Center of Lyon, 69424 Lyon, France; 10 Present Address: Institute of Biology and Molecular Pathology-CNR, 00161 Rome, Italy; 11 Present Address: Department of Medical Biochemistry and Biophysics, Karolinska Institutet, SciLifeLab, Stockholm, Sweden

**Keywords:** Cell signalling, Neural progenitors

## Abstract

Growth and patterning of the cerebellum is compromised if granule cell precursors do not properly expand and migrate. During embryonic and postnatal cerebellar development, the Hedgehog pathway tightly regulates granule cell progenitors to coordinate appropriate foliation and lobule formation. Indeed, granule cells impairment or defects in the Hedgehog signaling are associated with developmental, neurodegenerative and neoplastic disorders. So far, scant and inefficient cellular models have been available to study granule cell progenitors, *in vitro*. Here, we validated a new culture method to grow postnatal granule cell progenitors as hedgehog-dependent neurospheres with prolonged self-renewal and ability to differentiate into granule cells, under appropriate conditions. Taking advantage of this cellular model, we provide evidence that Ptch1-KO, but not the SMO-M2 mutation, supports constitutive and cell-autonomous activity of the hedgehog pathway.

## Introduction

The mammalian cerebellum represents a coordinating center where sensory inputs are integrated through an intricate and flexible set of neural connections to finely tune motor behaviors^1,2^, as well as cognitive and emotional spheres^3,4^.

In the mature cerebellum, different neuronal subtypes are distributed into distinct cortical layers that comprise: the molecular layer (ML), where the glutamatergic fibers of the granule cells (GC) synapse with dendrites of the Purkinje cells (PCs); the Purkinje cell layer (PCL), which contains the large somata of PCs; the internal granule cell layer (IGL) containing GCs bodies; and the innermost white matter (WM). PCs project to deep cerebellar nuclei (DCN), from which signals are then sent to the cerebral cortex.

GCs are the most numerous neurons of the brain, receiving and integrating sensory and motor inputs. Their remarkable role in cerebellar development and wiring is being progressively elucidated^5–12^. Importantly, growth and patterning of the cerebellum is compromised if GCs do not properly expand and migrate^8,10,13^. Their defects are associated with developmental disorders such as Joubert, Dandy-Walker and Nijmegen Breakage Syndromes; with neurodegenerative diseases such as Ataxia Telangiectasia and Spinocerebellar Ataxia, and with neoplastic diseases, such as medulloblastoma, or may manifest as autism and schizophrenia^14–16^ (see also https://medlineplus.gov/cerebellardisorders.html).

PCs and GCs derive from the ventricular zone (VZ) of the fourth ventricle and the rhombic lip (RL), respectively. Their reciprocal influences are essential for proper cerebellar foliation and lobule formation. In particular, Granule Cell Precursors (or progenitors, GCPs) undergo a first wave of expansion and migration from the RL toward the surface of the cerebellum, between E13.5 and E15.5, in mice, to populate the transient cerebellar External Granule Layer (EGL). A second proliferative wave occurs from postnatal (P) day 1 to 14, when GCPs exit the cell cycle and accumulate in the inner EGL, before they start their migration process to populate the IGL. During this phase, the surface area of the cerebellum increases much more than its volume due to the formation of up to ten lobules, in a process referred to as foliation. By P21 the EGL disappears, cerebellar lobes and foliation are complete, and the cerebellum is finally mature^9^.

Although multiple developmental pathways are involved in embryonic and postnatal cerebellar development, a prominent role is played by the Sonic Hedgehog (SHh) pathway. Secreted by the PCs, SHh drives the dramatic expansion of Atoh-1 positive GCPs to coordinate appropriate foliation^5,6,17^. The complex regulation of this pathway has been partially elucidated. In the absence of SHh, the 12-pass transmembrane receptor Patched 1 (Ptch1) constitutively inhibits the seven-pass transmembrane G protein coupled receptor Smoothened (Smo). This prevents the translocation of Smo to the primary cilium and keeps the pathway inactive. Under these conditions, Gli3, a member of the Gli family of transcription factors, becomes constitutively cleaved and converted into a transcriptional repressor. Moreover, the activity of Gli1-3 proteins is inhibited by Suppressor of Fused (Sufu), which regulates their cellular compartmentalization and proteolytic processing^18–22^. Upon binding of SHh to Ptch1, the inhibition on Smo is alleviated, allowing its accumulation at the primary cilium. Full-length Gli2 and Gli3 are released from Sufu, translocate to the nucleus to ignite a gene expression program^23^, which includes feed forward transcriptional activation of Gli1. How exactly Smo is constitutively activated, how Ptch-1 inhibits Smo and how Hh ligands relieve this inhibition are still incompletely solved questions.

Given the key role of GCs and the SHh pathway in cerebellar development and pathophysiology, studying proliferation, differentiation and migration of GCPs is pivotal to improve our understanding of cerebellar diseases and to provide new therapeutic chances. However, the paucity of appropriate cellular models to study GCs/GCPs *in vitro* still represents a major limitation. Three cell culture methods have been used so far, all presenting major disadvantages. Transient cultures of primary GCPs freshly explanted from (P5-P7) mouse cerebellum allow biomolecular investigation in a primary context, but only for few days, since they spontaneously differentiate into GCs in 5–7 days^24–26^. Hedgehog-type medulloblastoma cell lines, which are thought to originate from GCPs transformation, have also been used as surrogate for GCPs^27^. Not only the use of cancer cells to study GCPs pathophysiology is questionable, but common culture conditions for medulloblastoma cell lines quickly lead to Hh-pathway downregulation and gain of dependence on alternative pathways^28^. Finally, long term cultures of spheroids from postnatal cerebellar explants or medulloblastoma have been obtained using stem cell medium containing EGF and bFGF^29,30^. However, bFGF suppresses SHh pathway and impairs growth of GCPs^31^. All these cultures are therefore rather inappropriate to represent physiological GCPs.

New conditions to develop neurospheres tentatively of the GCP lineage have been recently proposed^32^. Here, we further defined this as a SHh-dependent GCPs model, which can grow as long-term primary neurospheres, undergo extensive self-renewal maintaining an active SHh pathway, and differentiate into GCs. Moreover, taking advantage of this cellular model, we addressed yet unclear steps in the SHh pathway by providing evidence that Ptch1-KO, but not the Trp535Leu mutation in SMO (SMO-M2), supports constitutive and cell-autonomous activity of the SHh pathway.

## Results

### Isolation and propagation of cerebellar GCPs with an active Hh-pathway

**Figure 1:**
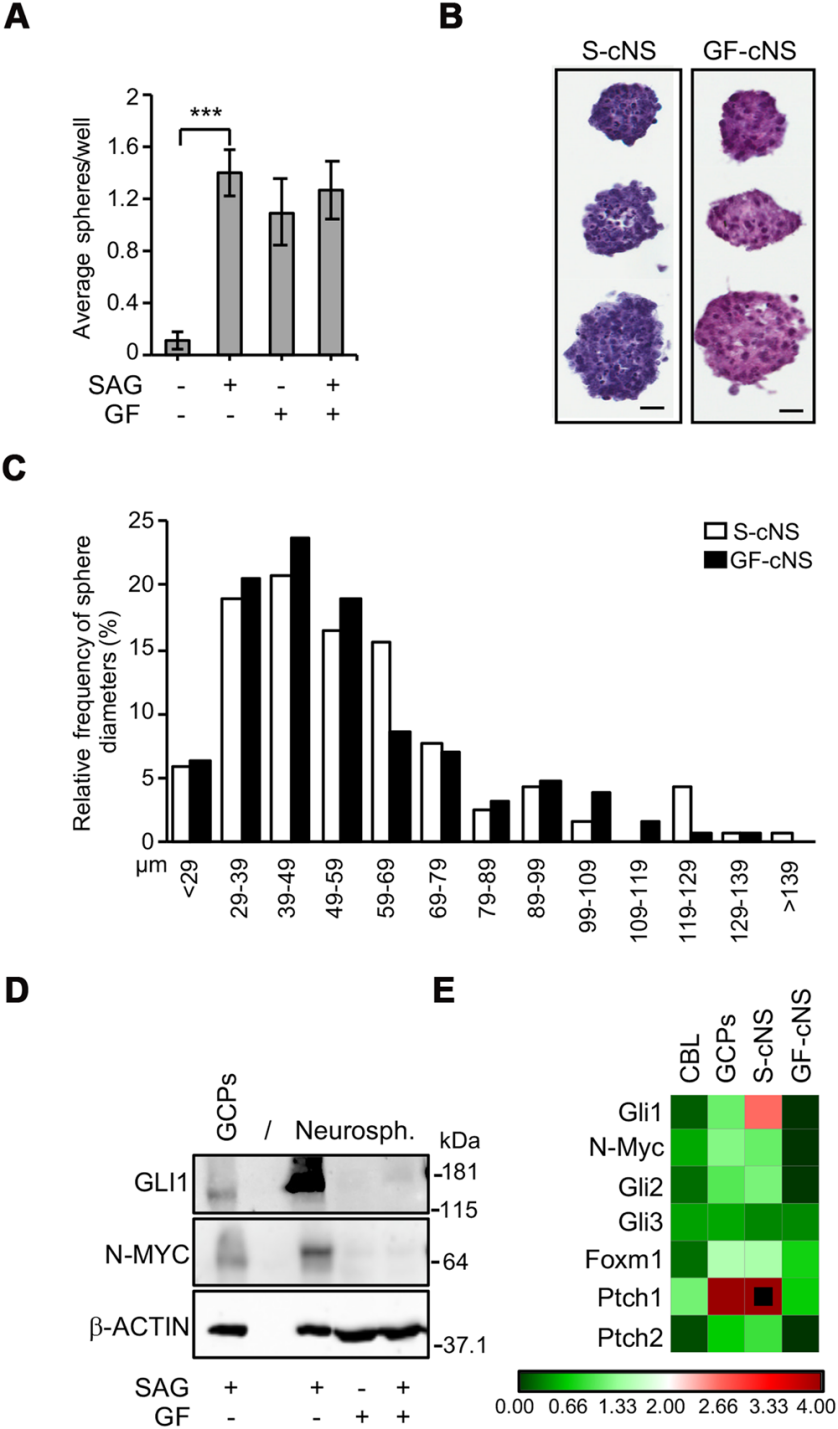
Generation of novel Hh-pathway active neurospheres from mouse cerebellum. (**A)** Neurosphere formation assay from P7 cerebellar explants grown in different conditions, as indicated. Data obtained by three independent experiments are reported as means +/− SD. (***p < 0.001). GF: EGF + bFGF cocktail. (**B**) Representative H&E staining of 1 week old S-cNS and GF-cNS of different sizes. Scale bar, 20 μm. (**C)** Frequency distribution analysis of the biggest diameter in S-cNS and GF-cNS ( ~100 spheres per sample). (**D**) Western Blot (WB) analysis comparing GLI1 and N-MYC protein expression in standard GCPs cultures and neurosphere cultures (grown as indicated), separated by an empty lane (indicated with/). Blots were probed for β-actin as a loading control. (**E**) RT-PCR quantification of known SHh target genes in P7 cerebellar extracts (CBL), standard GCPs cultures, S-cNS, and GF-cNS. We used three independent samples for each experimental condition. Transcripts expression was normalized on the mean expression level of four reference genes: Pgk1, Hprt, Gusb, Tfrc. Black dots: fold changes exceeding the maximum value of the given scale. Uncropped Western blot images related to this figure are displayed in Fig. S7.

Explants from mice cerebella at P5-7 are commonly used to establish SHh-stimulated short-term GCP cultures^24–26^ or long term neurospheres grown in “stem cell medium” containing a EGF/bFGF cocktail (from now on: GF)^29,30^. However, bFGF suppresses SHh activity, preventing GCPs expansion *in vitro*^31^, suggesting that these conditions are not appropriate to propagate cells with active Hh signaling. In contrast, Ptch1 loss allows growth factor-independent expansion of medulloblastoma cells^33^, and stimulation of the Hh-pathway by the Smo agonist SAG suffices to establish long-term cerebellar neurospheres^32^. Thus, we further and extensively characterized SAG-dependent cerebellar neurospheres (from now on S-cNS) in comparison with GF-dependent cerebellar neurospheres (from now on GF-cNS). As expected, GF, SAG or GF + SAG all promoted clonogenic growth of neurospheres from P7 cerebellar explants, while these did not form in the absence of mitogenic stimuli (Fig. 1A). Mean size (57.83 vs 55.24 μm), median size (52.59 vs 49.00 μm) and range (21.77–141.37 vs 20.81–135.57 μm) of S-cNS and GF-cNS were similar, although S-cNS showed a greater nuclear density (Fig. 1B,C). The expression analysis of Hh-dependent biomarkers at the protein (GLI1 and N-MYC) and mRNA (Gli1/2, N-Myc, FoxM1 and Patch1/2 transcripts) levels revealed that S-cNS, but not GF-cNS, were characterized by sustained activation of the Hh-pathway, similar to short-term GCPs cultures (Fig. 1D,E). The lack of GLI1 and N-MYC protein expression in neurospheres grown with SAG + GF (Fig. 1D) and the low levels of Hh-dependent transcripts in GF-cNS compared to short term GCPs and S-cNS (Fig. 1E) confirmed that bFGF suppresses Hh activity, supporting previous findings^31^. SAG concentrations ranging from 0.1 to 1 μM allowed clonogenic growth of Hh-active cells, being 0.2 μM the lowest and most effective concentration (Fig. S1A,B), consistent with the notion that excessive SAG generates inhibitory signals on short-term GCPs cultures^34^.

**Figure 2:**
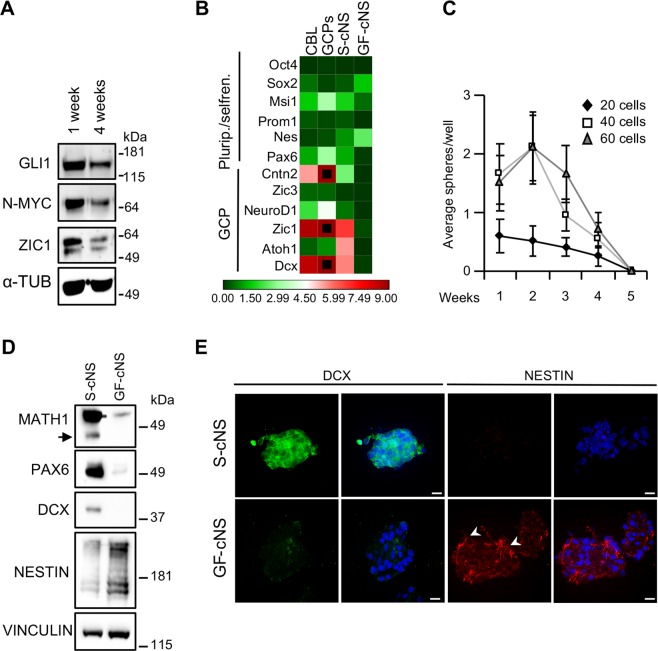
S-cNS are self-renewing cultures expressing GCP markers and Hh-pathway activity. (**A**) WB analysis of the indicated proteins, in 1 and 4 weeks old S-cNS. Blots were probed for α -tubulin as a loading control. (**B**) RT-PCR quantification of the indicated transcripts. We used three independent samples for each experimental condition. Transcripts expression was normalized on the mean expression level of four reference genes: Pgk1, Hprt, Gusb, Tfrc. Black dots: fold changes exceeding the maximum value of the given scale. (**C**) Neurosphere formation assay obtained by repeatedly dissociating S-cNS and reseeding a different number (20, 40, 60) of cells/well every week, to test self-renewal properties over a period of 5 weeks. Data obtained by three independent experiments are reported as means +/− SD. (**D**) WB analysis of the indicated proteins in S-cNS compared to GF-cNS. Blots were probed for vinculin as a loading control. (**E)** Immunostaining of S-cNS and GF-cNS neurospheres for DCX (green) and NESTIN (red). Cell nuclei are evidenced (in blue) by HOECHST- staining. The arrows point out typical NESTIN filaments. Scale bar, 10 μm. Uncropped Western blot images related to this figure are displayed in Suppl. Fig. 8.

A key feature of stem and progenitor cells is their ability to undergo self-renewal. Importantly, S-cNS could be repeatedly dissociated and re-seeded in clonogenic conditions allowing propagation for more than 4 weeks with an active Hh-pathway, indicated by GLI1 and N-MYC expression (Fig. 2A). Moreover, cells in S-cNS were capable of EdU incorporation in a large proportion (Fig. S2A), and maintained high expression of proliferation and metabolic markers (Fig. S2B) and a relevant expression of progenitor/self-renewal markers, most prominently Msi1, as in freshly explanted GCPs (Fig. 2B). Varying from GF-cNS, which may be grown indefinitely, in time we observed a gradual decrease of S-cNS self-renewal capability (Fig. 2C), with only minor changes in the expression of Hh-pathway and general gene expression pattern (Figs. 2A, S2D), suggesting a senescence-like crisis. Occasionally S-cNS spontaneously recovered after this crisis and eventually proliferated indefinitely.

Interestingly, the overall expression profile was rather different between S-cNS and GF-cNS, suggesting major differences in the identity of the two populations of cerebellar neurospheres^31^. In particular, S-cNS expressed higher levels of the GCP markers Atoh1, Pax6, Dcx, Cntn2, Zic1 and NeuroD1 compared to GF-cNS (Fig. 2B), while Sox2, Nestin, Olig1/2 and Cd81 were more expressed in GF-cNS than in S-cNS (Figs. 2B and S2C). Consistently, S-cNS showed a high expression of ATOH1, PAX6 and DCX proteins (Fig. 2D), the last showing a diffused expression in most cells of each neurosphere (Fig. 2E). In contrast, GF-cNS showed a high expression of Nestin1 (Fig. 2D), which was detectable in many cells of each neurosphere, as indicated by immunofluorescence analysis (Fig. 2E). Scant Nestin1 expression was detectable in S-cNS (Fig. 2D,E). A very modest expression of differentiation markers was detectable on both neurospheres cultures (Fig. S2D).

**Figure 3:**
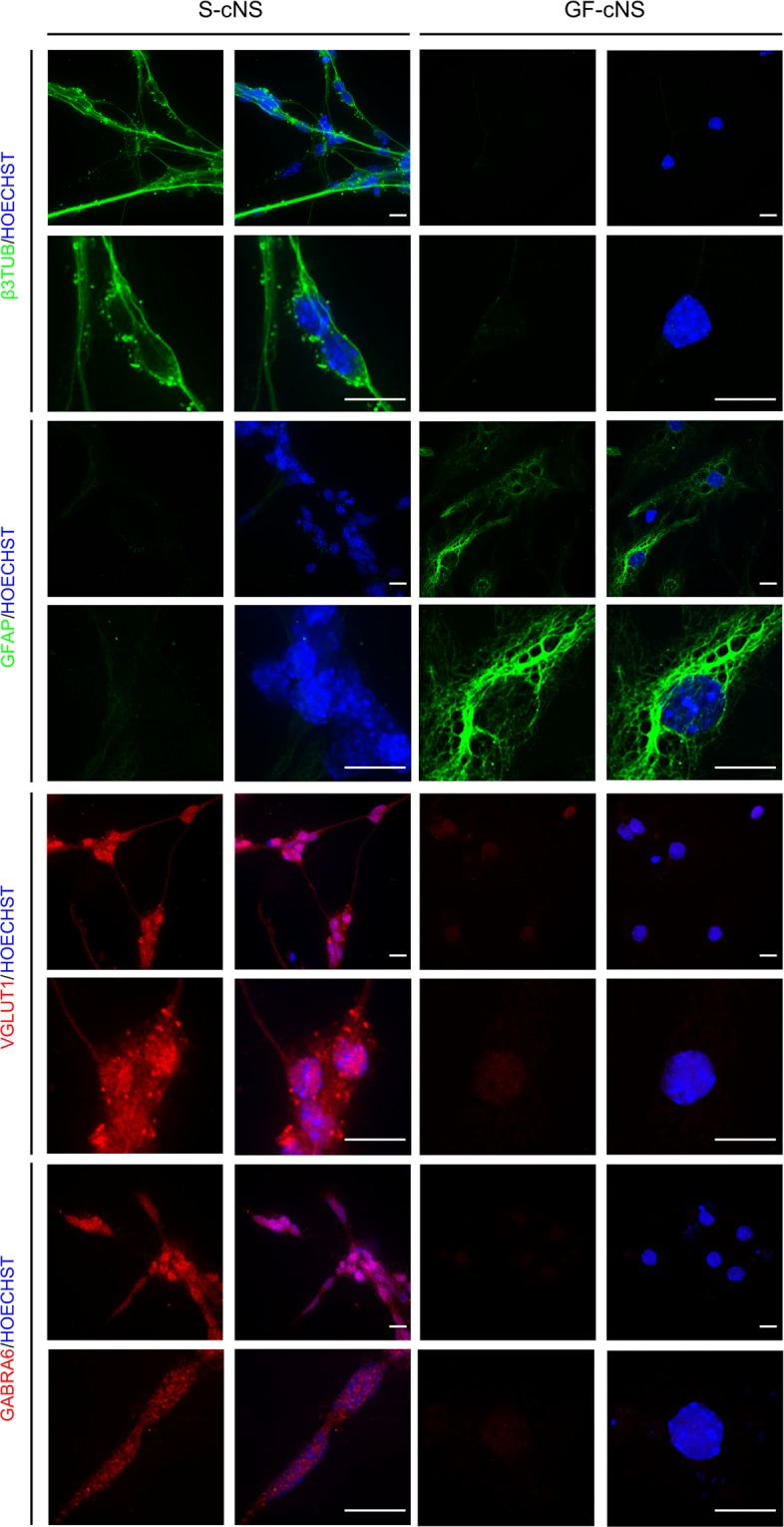
Adherent S-cNS-derived cells, but not GF-derived cells, differentiate into GCs in appropriate conditions. Immunostaining for β3-TUBULIN (green), GFAP (green), VGLUT1 (red) and GABRA6 (red) proteins in S-cNS and GF-cNS -derived cells grown onto polylysine-coated substrates, and treated with differentiation medium containing FBS + VitA, for 96 hours. The images are representative of three independent experiments. Cell nuclei are evidenced (in blue) by HOECHST- staining. Scale bar, 10 μm.

GCPs differentiate into GC in adherent cultures. To test whether S-cNS accounted for precursors of granule cells, we seeded dissociated neurospheres on a poly-lysine coated substrate and cultured them with fetal bovine serum (FBS) with or without vitamin A (VitA), to address their differentiation properties^35^. S-cNS-derived cells treated with FBS and FBS + VitA displayed the typical neuron-like morphology characterized by a fusiform cell body extending few neurites of different lengths and establishing connections with other cells (Figs. 3 and S3). Moreover, the vast majority of them expressed the neuronal marker β3-tubulin and GABRA6 and VGLUT1 proteins (Figs. 3 and S3), suggesting their differentiation into GCs^36,37^. Only occasionally, star-shaped and GFAP positive cells could be detected in S-cNS-derived adherent cells cultured in differentiation media (Fig. S3). By contrast, a vast majority of adherent GF-cNS-derived cells grown in differentiation media acquired a star-shaped GFAP-positive phenotype and only occasionally showed a β3-tubulin-positive neuron-like appearance, suggesting a glial determination (Figs. 3 and S3). Moreover, they were fully negative for GABRA6 and VGLUT1 proteins.

Overall, these data indicate that S-cNS represent a novel long-term and nearly homogeneous culture of proliferating precursors of granule cells (likely GCPs) maintaining an active Hh-pathway and the ability to differentiate into GCs.

### S-cNS are continuously dependent on Hh-pathway for survival and proliferation

**Figure 4:**
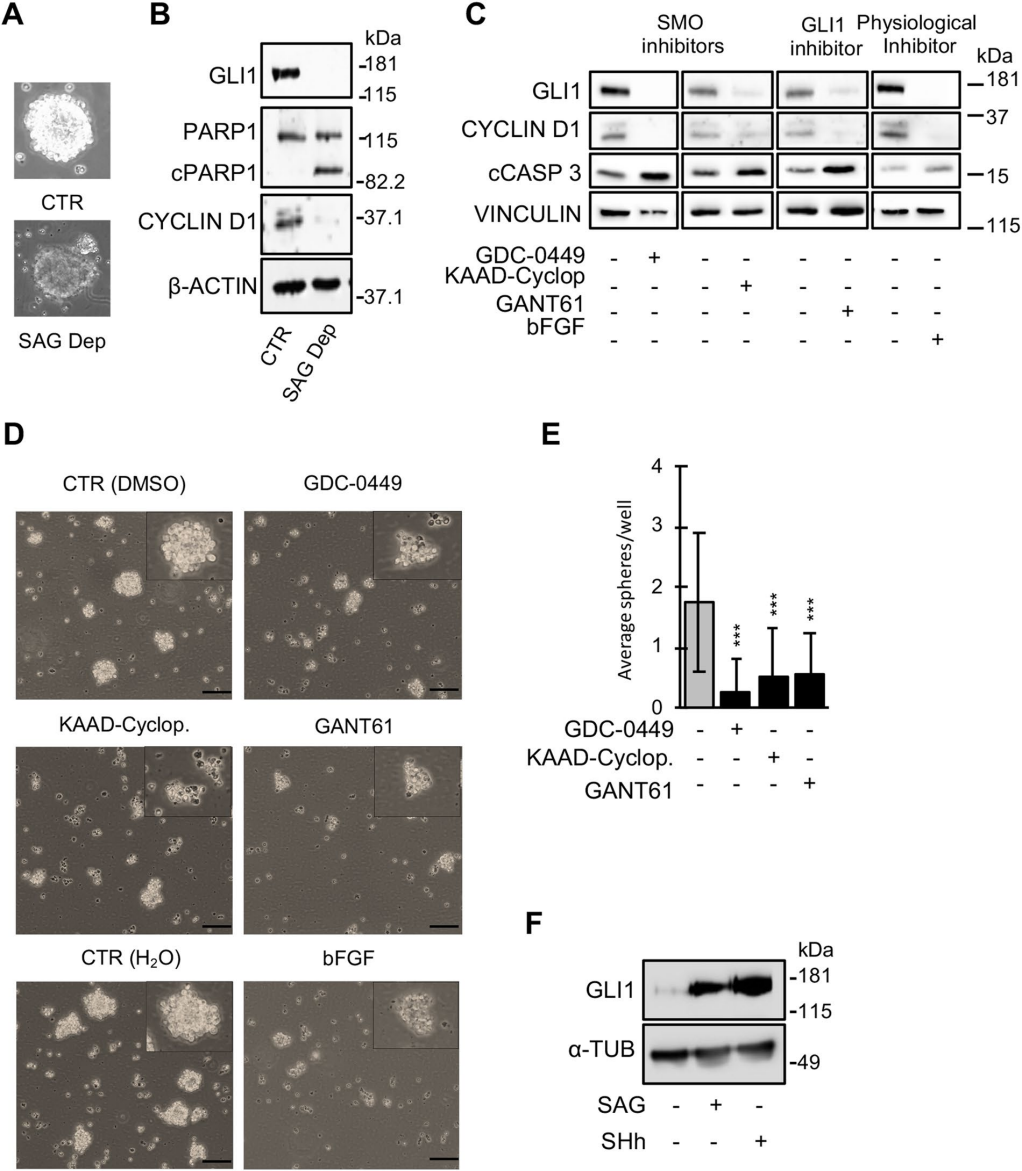
S-cNS remain dependent on the SHh pathway for growth/survival in time. Two weeks old S-cNS were used for this set of experiments. (**A**) Representative contrast microphotographs and (**B**) WB analysis of the indicated proteins in control or SAG-deprived (24 hours) S-cNSs. Blots were probed for β-actin as a loading control. (**C**) WB analysis of the indicated proteins and (**D**) representative contrast pictures of S-cNS after exposure to the specified Hh-pathway pharmacological (GDC-0449, KAAD-cyclopamine and GANT61) or physiological (bFGF) inhibitors (scale bar 70 μm). Blots were probed for vinculin as a loading control. DMSO or water were used as mock controls for pharmacological inhibitors or bFGF, respectively. Inserts show higher magnitude pictures of single spheres. (**E**) Neurosphere formation assay of S-cNS exposed to Hh-pathway inhibitors. (**F**) WB analysis of GLI1 expression in S-cNS from which SAG was washed out and immediately replaced with medium containing either DMSO (as a control), SAG, or N-terminal recombinant SHh, for 24 hours. GLI1 expression declines after 24 hours of SAG deprivation. Instead, its expression remains equally sustained in cultures re-stimulated with SHh or SAG. Blots were probed for α -tubulin as a loading control. Uncropped WB images related to this figure are displayed in Suppl. Figs. 9 and 10.

To evaluate whether S-cNS remained strictly dependent on Hh-pathway for survival and proliferation, we withdrew SAG in the absence of differentiation inputs. SAG washout quickly caused cell death, as indicated by the conversion of the bright and translucent appearance of S-cNS into dark and opaque spheres containing pyknotic cells (Fig. 4A). Consistently, 24 hours of SAG deprivation were sufficient to abrogate the expression of Hh targets GLI1 and CYCLIN-D1 and to allow PARP1 cleavage, suggesting a complete shut-off of the pathway and the occurrence of cell death by apoptosis (Fig. 4B).

To further exclude the possibility that S-cNS could be dependent on Hh-unrelated off-target effects of SAG, we directly ablated Hh pathway by chemical inhibitors operating at different levels in the Hh-pathway, such as KAAD-cyclopamine, GDC-0449 and GANT61 or by a physiological inhibitor (bFGF). All chemical inhibitors down-regulated GLI1 and CYCLIND1 expression, reduced spheroid size and impaired S-cNS survival and self-renewal (Figs. 4C–E and S4), indicating a true addiction of these cells to the Hh-pathway. Similar data were obtained with bFGF (Fig. 4C,D).

To test whether SHh could substitute for SAG in maintaining Hh-pathway activity, we removed SAG from S-cNS and replaced it with N-terminal recombinant SHh. While SAG removal led to GLI1 suppression, its replacement with SHh restored GLI1 expression as efficiently as SAG restoration (Fig. 4F).

Overall our data indicate that constitutive activation of the Hh-pathway (i.e. via SAG) is necessary and sufficient for the genesis and *in vitro* maintenance of a nearly homogeneous population of non-transformed GCPs.

### Generation of S-cNS can be accomplished from cerebellar explants containing proliferating GCPs

During postnatal cerebellar development, the population of GCPs quickly expands in the EGL before starting migration to the IGL and differentiation into mature GCs. This proliferation phase starts at P1, peaks at P5/P7 and is roughly concluded around P14 and, by P21, foliation and differentiation into mature GCs are completely accomplished (Fig. S5A). Based on this, we tested the possibility to generate S-cNS from cerebellar explants from P1 to P21. Interestingly, we managed to obtain S-cNS from P1 and P7 explants, while we only occasionally obtained few neurospheres at P14 and P21 (Fig. S5B), likely suggesting that SAG cannot recruit post-mitotic and mature GCs into sphere formation.

### Ptch1 deletion, but not the SMO-M2 mutation, induces cell autonomous growth of cNS

**Figure 5:**
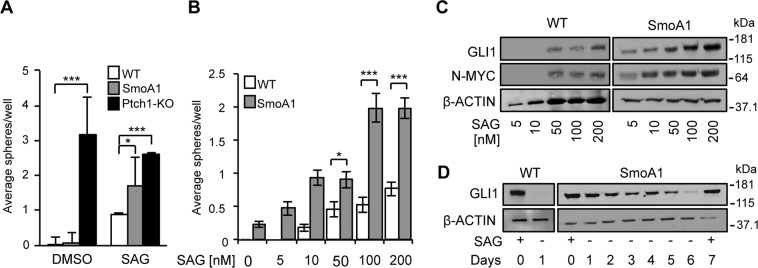
Cerebellar explants from Ptch1-KO, but not SmoA1, mice allow generation of neurospheres without SAG stimulation. (**A**) Neurosphere formation assay on P7 WT, SmoA1 and Ptch1-KO cerebellar explants with or without SAG. Data obtained by three independent experiments are reported as means +/− SD. (*p < 0.05; ***p < 0.001). (**B**) Neurosphere formation assay on P7 WT and SmoA1 cerebellar explants, with different SAG concentrations. Data obtained by three independent experiments are reported as means +/− SD. (*p < 0.05; ***p < 0.001). (**C**) WB analysis of the indicated proteins in S-cNS cultures derived from the same explants and then treated as in (**B**). (**D**) WB analysis of GLI1 expression in WT and SmoA1 S-cNS exposed to SAG, or SAG-deprivation (SAG−) and collected at the indicated time points. Blots were probed for β-actin as a loading control. Uncropped Western blot images related to this figure are displayed in Suppl. Fig. 11.

Early conditional knock-out of the Ptch1 inhibitory receptor in animal models leads to constitutive activation of the Hh-signaling, which promotes derangement in cerebellar development and medulloblastoma^38^ (Fig. S5A). Also the Neuro D2-driven expression of the SMO-M2 mutation in the SmoA1 mouse model induces medulloblastoma, due to a constitutive activation of the Hh-pathway^39,40^. However, only a transient enlargement of the EGL can be detected in the SmoA1 mice^40^ rather than the dramatic derangement of postnatal cerebellar development seen in the Ptch1-KO model^38^ (Fig. S5A), suggesting that Ptch1 deletion and SMO-M2 mutation do not fully overlap in functional terms. Since SAG-induced Hh-pathway is sufficient to drive clonogenic neurosphere formation from P1-P7 WT cerebellar explants, we reasoned that cerebellar explants from the Math-CRE/Ptch1^C/C^ (from now on Ptch1-KO) and SmoA1 mouse models should be able to give origin to neurospheres even in the absence of SAG. Consistently, we could efficiently generate Ptch1-KO neurospheres either in the presence or absence of SAG (Fig. 5A and Table 1), confirming that Ptch1 loss leads to constitutive activation of the Hh-pathway and a “cell autonomous” growth of cerebellar neurospheres (cNS), in the absence of further mitogenic stimuli. In sharp contrast, explants from the SmoA1 mouse were not competent for growth and survival in the absence of SAG, which suggests that the SMO-M2 mutation was not sufficient to activate Hh-pathway in a cell autonomous context (Fig. 5A). Although they never reached Ptch1-KO scores, SmoA1 explants appeared more efficient in the generation of S-cNS than WT explants. Indeed, lower amounts of SAG supported the clonogenic growth of neurospheres and the induction of GLI1 and N-MYC from SmoA1 compared to WT explants (Fig. 5B,C and Table 1). Moreover, SmoA1 S-cNS undergoing SAG deprivation experienced shut-off of the Hh-pathway and cell death at much later times compared to WT S-cNS (Fig. 5D).

**Table 1:** Summary of the developmental stages allowing generation of S-cNS from WT, SmoA1 and Ptch1-KO cerebella.

Animal strain	P1	P7	P14	P21	
WT	+	+	−	−	+SAG
SmoA1	+	+	+	−	
Ptch1-KO	+	+	+	+	
WT	−	−	−	−	−SAG
SmoA1	−	−	−	−	
Ptch1-KO	+	+	+	−	

Noteworthy, the inability to grow in the absence of SAG did not depend on the loss or attenuation of the expression of the Smo transgene in S-cNS. Rather, they showed higher levels compared to P7 cerebellar extracts from SmoA1 animals (Fig. S6).

Thus, Ptch1-KO, but not SMO-M2 mutation, confers constitutive and cell autonomous activation of the Hh-pathway under our neurosphere formation assay.

### Hh-active cNS generated via different approaches share a very similar gene expression profile

**Figure 6:**
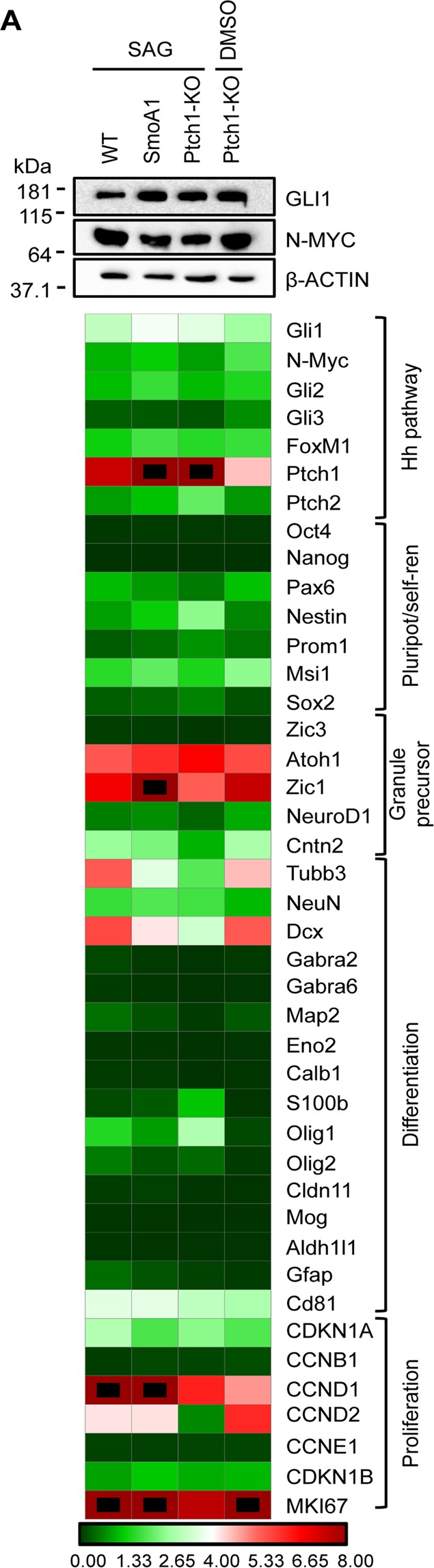
Hh-active neurospheres share a very similar gene expression profile. WB analysis of the indicated proteins and RT-PCR quantification of the indicated transcripts in 2 weeks old WT, SmoA1 and Ptch1-KO S-cNS and Ptch1-KO cNS. Blot was probed for β-actin as a loading control. For RT-PCR quantification, we used three independent samples for each experimental condition. Transcripts expression was normalized on the mean expression level of four reference genes: Pgk1, Hprt, Gusb, Tfrc. Black dots: fold changes exceeding the maximum value of the given scale. Uncropped Western blot images related to this figure are displayed in Suppl. Fig. 12.

Of interest, the 4 sets of neurospheres generated from WT, SmoA1 and Ptch1-KO mice (+/− SAG) shared a very similar expression of a number of relevant genes (Fig. 6). They all showed very low expression of neuronal or glial differentiation markers, and high expression of the GCPs markers Atoh1, Zic1, Pax6 and Dcx, and cell cycle related genes (including cyclins, MKI67, E2F1). As expected, they all showed relevant expression of Hh target genes, including Gli1 and Gli2, N-Myc, FoxM1, and Ptch1.

Overall, these data indicate that the Smo-dependent activation of the Hh pathway, either due to Ptch1-KO or SAG administration, promotes the growth of very similar Hh-active GCP neurospheres from P7 cerebellar explants.

### Both Ptch1-KO and SMO-M2 mutation prolong the possibility to generate Hh-active cNS from cerebellar explants

**Figure 7:**
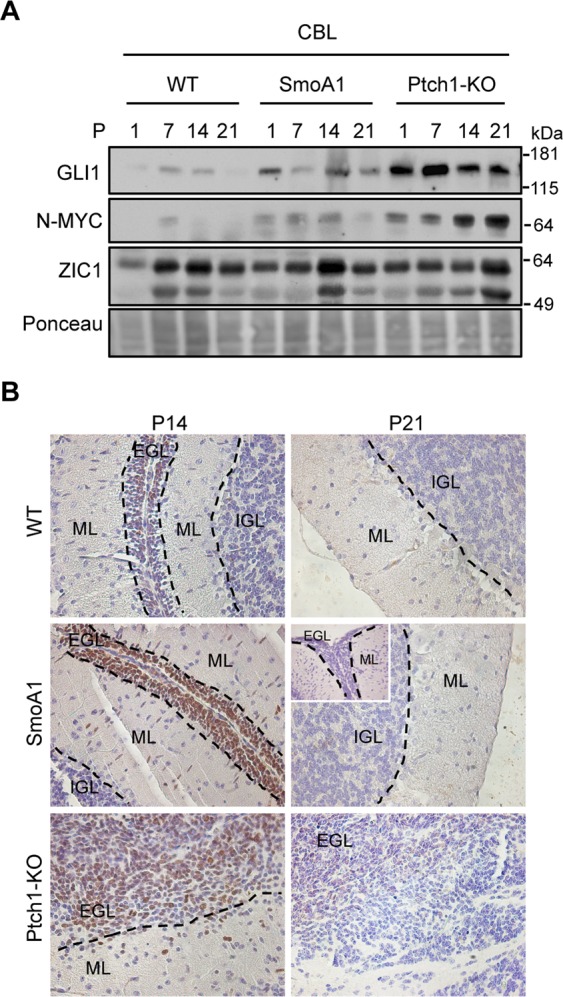
Different features of SmoA1 and Ptch1-KO mouse models during postnatal cerebellar development. (**A**) WB analysis of the indicated proteins in total extracts from WT, SmoA1 and Ptch1-KO cerebella, collected at different stages of post-natal development. Ponceau staining was used as a loading control. (**B**) Sagittal sections from P14 and P21 WT, SmoA1 and Ptch1-KO mouse cerebella were analyzed for Ki67 expression by immunohistochemistry (magnification 20X). The inset shows in greater detail an area containing a high concentration of resting cells, which are negative for Ki67 immunoreactivity, in a representative region of P21 SmoA1 cerebella. EGL: External Granular Layer; ML: Molecular Layer; IGL: Internal Granular layer. Uncropped Western blot images related to this figure are displayed in Suppl. Fig. 13.

Considering GLI1 and N-MYC expression as reporters of Hh activity indicates that both SMO-M2 mutation and Ptch1-KO extend the window of activation of the Hh-pathway compared to WT mice during cerebellar postnatal development (Fig. 7A), as previously shown^38,40^. In particular, while GLI1 and N-MYC expression sharply decline between P7 and P14 in WT cerebella, we can promptly detect their expression up to P14 and to P21 in SmoA1 and Ptch1-KO cerebella, respectively (Fig. 7A). Consistently, we found a much higher percentage of Ki67-positive GPCs in p14 SmoA1 and Ptch1-KO cerebella compared to WT, while only Ptch1-KO cerebella still showed Ki67 labelling at p21 (Fig. 7B). Indeed, even the nests of “resting” GCPs that can be frequently observed in SmoA1 cerebella at P21 stain negative for Ki67 (Fig. 7B inset). Importantly, a time dependent accumulation of mature and non-proliferating GCs occurs in all models, with WT and SmoA1 cerebella becoming nearly identical at P21 (Fig. S5A).

Interestingly, we were able to generate long-term neurospheres from both SmoA1 and Ptch1-KO P14 explants, but not from P14 WT, and P21 WT and SmoA1 explants (Table 1), consistent with the idea that Hh-active cNS could be generated only from proliferating GCPs. Intriguingly, we were able to generate S-cNS from P21 Ptch1-KO explants (Table 1), which still contain relevant amounts of Ki67 positive GCPs in age-matched cerebellar sections (Fig. 7B), while we repeatedly failed to generate spontaneous cNS even from this model, at this specific time.

## Discussion

A multiplicity of animal models has been used to characterize the role of GCs in cerebellar development and disease. In contrast, a short-term culture of primary GCPs^24–26^ has been so far the only valid tool to address biological/biochemical issues related to GC pathophysiology, *in vitro*. Even though this model allowed a number of studies on GCPs and on the pathways governing their proliferation, it can only be used for short term assays, since its Hh-responsiveness quickly declines and cells terminally differentiate into GCs in just a few days.

Making use of a recently published method^32^, here we validated culture conditions to grow freshly explanted cerebellar GCPs in the form of neurospheres, supported by SAG-dependent activation of the Hh-pathway, in the absence of additional GF, which we named S-cNS. Significantly adding to the previously mentioned publication, we show these cells: (i) show prolonged self-renewal and expression of progenitor markers; (ii) maintain an active Hh-pathway, from which they depend on for proliferation and survival; (iii) can differentiate into GCs under defined conditions; (iv) can be used to address issues related to SHh signal transduction, and, in principle, any other GCPs/GCs biological and biochemical issue, in a native and cell autonomous context.

At variance with previous observations^32^, S-cNS show a long, but limited, lifespan, which is consistent with their “progenitor” nature. Rarely, after a senescence-like crisis, few proliferating spheroids emerged and could be grown for longer times (not shown), but their biological features have not been fully characterized yet. Implementing previous observations^32^, we provide a much more extensive characterization of the S-cNS identity. The expression of multiple GCP markers, the activation of Hh-dependent targets and the proliferation-high/differentiation-low gene expression signature identify these cells as proliferating, Hh-responsive GCPs^11,23,41–43^. This is further confirmed by their ability to differentiate into β3-tubulin-positive neurons expressing typical GC markers, such as VGLUT1 and GABRA6^36,37^.

The modest, yet detectable expression of NESTIN in S-cNS raises the question of their possible origin from two distinct populations of Hh-responsive Nestin-expressing progenitors (NEP) described by Li *et al*.^44^ and Wojcinski *et al*.^11^, both of which may give rise to GCs, *in vivo*. While we cannot formally exclude that S-cNS may also include NEP-deriving cells, we consider this unlikely and favor the hypothesis that S-cNS are more likely GCPs. Indeed, Atoh-1-negative NEPs described by Li *et al*. are rare cells representing no more than 3–5% of the EGL^44^, while Atoh-1 GCPs are the most abundant cell population in P7 cerebella. NEPs described by Wojcinski *et al*. are white matter-derived pluripotent cells that can differentiate in multiple lineages^11^. Thus, both NEPs seem rather different from our S-cNS that are Atoh-1 positive and show commitment to GC differentiation. Most important, S-cNS dramatically differ from GF-cNS, which are more likely to reflect the FGF-responsive non-granule cell precursors described by Lee *et al*.^29^.

Along the 4–5 weeks of self-renewal, the S-cNS remained untransformed and also responded to physiological inhibitory signals, such SAG removal or bFGF. Indeed, inhibition of the Hh-pathway in S-cNS resulted in very quick death, unless differentiation conditions were implemented. This supports the strong and continuous dependence of S-cNS from Hh-signaling, a feature frequently lost by culturing Hh-dependent medulloblastoma cells in FBS-containing media^28^.

Very similar S-cNS could be obtained from WT, SmoA1 and Ptch1-KO mice, until proliferating GCPs could be detected in the cerebella of each animal model (P7, P14 and P21, respectively). The possibility to grow Ptch1-KO spheres in the absence of SAG and any other GF unequivocally demonstrates that activation of the Hh-pathway is the only requirement for GCPs expansion *in vivo* and *in vitro*. Of interest, SAG-dependent, but not SAG-independent neurospheres, could be generated from Ptch1-KO mice at P21. Although still unclear at the moment, this piece of data seems to suggest that a Ptch1-independent inhibition of Smo might be occurring in Ptch1-KO P21 GCPs, which can be relieved by SAG administration. Whether this is relevant to the yet undefined mechanism that leads to the differentiation of GCs in Ptch1-KOs despite Hh activity^38^ still remains to be clarified.

Here, we used S-cNS to address the still debated question of whether the SMO-M2 mutant is really constitutively active and no more inhibitable by the Ptch1 receptor. This mutation, originally described in human basal cell carcinoma^45^, appears to fully activate the Hh-pathway in different functional assays, if exogenously expressed, as if it was constitutively active and insensitive to Ptch inhibition^34,46^. Moreover, its transgenic expression in mice leads to basal cell carcinoma and medulloblastoma formation^39,40,45^. In principle, loss of the inhibitory action of Ptch1 or constitutively active Smo should lead to identical consequences. However, while SmoA1 and Ptch1-KO animal models are both characterized by highly penetrant medulloblastoma development, the phenotypes of the two do not fully overlap. Indeed, all Ptch1-KO mice are affected by clinically evident medulloblastoma between 10–12 weeks, while a certain variability in frequency and age of onset, dependent on the copy number of the transgene and on the genetic background, characterize the SmoA1 model^38–40^. Moreover, the SMO-M2 transgene has a less dramatic effect on cerebellar postnatal development, with only transient enlargement of the EGL and persistence of scattered hyperplastic lesions compared to Ptch-1 KOs, which are characterized by extensive derangement of cerebellar cytoarchitecture that progressively transforms into full blown tumor lesions^38,40^. S-cNS cultures confirmed the existence of major differences between WT and mutant Smo conditions, but also revealed that Ptch1-KO, but not SMO-M2 mutant, is sufficient to maintain a constitutive Hh activity and to support GCP survival/proliferation, in a cell autonomous context. Intriguingly, Ptch1-KO explants also provided the highest performances in the clonogenic assay compared to SAG-induced WT or SmoA1 explants. Although not investigated in this paper, the occurrence of Gli-independent non-canonical Hh signaling ignited by Ptch1 inhibition (or loss), but independent from canonical Smo signaling^47–49^, should be taken into account when considering these data. Also, a nuclear localization of SMO, which drives GLI1 activation and is unresponsive to SMO inhibitors, has been reported to occur in Ptch1-silenced Basal Cell Carcinoma cells, again suggesting the activity of alternative non-canonical Hh-signaling^50^. Further work will be required to shed more light on this issue.

Our data seem to indicate that Ptch-1 may exert at least a partial inhibition on SMO-M2, which may be released by SAG administration. As a matter of fact, the mechanisms of constitutive Smo activity and the inhibition by Ptch-1 are still incompletely understood, as is the mechanistic role of Smo oncogenic mutations^51^. Accumulating evidences indicate that unliganded Ptch-1 would catalytically regulate the access of an endogenous small molecule to Smo^52^. Similar to other G-protein coupled receptors, Smo appears to oscillate between an active and an inactive conformation and cholesterol is gaining momentum as the putative endogenous activator of Smo^53,54^. A hydrophobic tunnel in the seventh transmembrane domain would allow cholesterol to ascend towards the cysteine-rich domain, in the active Smo conformation^55^. This tunnel can be closed, and Smo inactivated, by an “ionic lock” involving residues W535 on TM7 and R451 on TM6. Interestingly, the W535L SMO-M2 mutation seems to ablate the ionic lock, blocking Smo in the active conformation^55^. According to our data, however, the SMO-M2 mutation is not sufficient to allow cell autonomous Hh-pathway activation and cNS growth, suggesting that the locked active conformation conferred by the W535L change is likely to be still at least partially inhibitable by Ptch1. A hypothesis reconciling all available data is that, due to its structural constraints, the SMO-M2 mutant is more prone to be released from Ptch-1 inhibition in the presence of SHh, leading to prolonged Hh signaling and expansion of proliferating pools during cerebellar (or skin) development. These conditions might potentially lead to the hyperplastic lesions occurring in the SmoA1 mouse, from which transformation could take place, upon additional molecular events.

In conclusion we believe that S-cNS is a flexible and handy tool useful to the scientific community to study GCPs/GCs properties *in vitro*, and an excellent device to test the activity of physiological or chemical SHh inhibitors. As we have shown here, it also represents and invaluable tool to address issues related to SHh signaling in a native GCP context.

## Experimental Procedures

### Animal models

The Math1-Cre/Patch1^C/C^ mice have been previously described^38^ and each strain was purchased from The Jackson Laboratory. The SmoA1 mice have been previously described^39^ and were kindly given by Prof. James M. Olson, Clinical Research Division, Fred Hutchinson Cancer Research Center, Seattle, Washington.

Animal use was approved by the Italian Ministry of Health (protocol n°379/2016-PR) and was performed according to the guidelines for animal care.

### Establishment of standard GCPs and GCP-neurosphere cultures from murine cerebellum

GCPs were isolated and grown as described^56^. Briefly, mice cerebella were removed aseptically, cut into small pieces, incubated for 15 min in digestion buffer (PBS/5 mM EDTA, plus 0.3 U/ml DNase, Sigma, St. Louis, MO), at room temperature. After grinding tissue in PBS/5% FBS/0.3 U/ml DNase using pipettes of decreasing bore size to obtain a single-cell suspension, cells were resuspended in Neurobasal medium (Invitrogen) supplemented with B27 + VitA (Life Technologies), penicillin-streptomycin 1%, glutamine 1% (Sigma Aldrich G7513) and 5% FBS (Invitrogen) and seeded in polylysine-coated dishes. After 3 hours, medium was replaced with fresh complete Neurobasal (w/o FBS) supplemented with 200 nM SAG (Adipogene).

For GCP-neurosphere cultures, explanted cerebella were collected in HBBS (GIBCO) supplemented with 0.5% glucose and penicillin-streptomycin (Sigma Aldrich), grossly grinded with a serological pipette and treated with DNAse I to a final concentration of 1.28 U/ml for 30’. Cell aggregates were mechanically dissociated to obtain single-cell suspensions. After centrifugation, cells were seeded in selective medium: DMEM/F12 (GIBCO) supplemented wih 0.6% glucose, 25μg/ml insulin (Sigma Aldrich), 60μg/ml N-acetyl-L-cysteine (Santa Cruz), 2μg/ml heparin (Sigma Aldrich), penicillin-streptomycin and B27 supplement without VitA (Life Technlogies). SAG was used at the concentration of 200 nM, unless otherwise specified. When indicated, bFGF and EGF (Peprotech) were added to a final concentration of 1 μM.

Whenever necessary, neurosphere cultures were pelleted and dissociated by incubation with Accutase to obtain a single cell suspension.

### Cell treatments

For neurosphere forming assays, after dissociation cells were seeded at 20 cells/well, in 96 well plates (unless otherwise specified) in the appropriate medium. Medium was eventually replenished every 3–4 days. The number of spheres/well was counted after 1–2 weeks.

For drug treatments, after dissociation, cells were seeded at 50.000 cells/cm^2^ and treated with SAG, with or without KADD-cyclopamine (100 nM, Calbiochem), GDC-0449 (0.5 μM, Selleckchem), GANT61 (5 μM, Enzo Lifesciences) and bFGF (1 μM, PeproTech). Neurospheres were harvested after 48 (GDC-0449 and bFGF) or 96 hours (KAAD-cyclopamine and GANT61) for further analyses.

To evaluate the responsiveness to SHh ligand, after dissociation cells were seeded at 50.000 cells/cm^2^ and supplemented with SAG. After 24 hours, SAG was removed by washing in PBS and replaced with N-terminal recombinant SHh (3 μg/ml, R&D Systems), SAG or nothing. Neurospheres cultures were harvested after 72 hours for further analyses.

For differentiation experiments, dissociated cells were plated onto polylysine-coated dishes at 125.000 cells/cm^2^ in the following medium: Neurobasal (Invitrogen) supplemented with B27 (Life Technologies, #12587010), 0.6% glucose, 25 μg/ml insulin, 60 μg/ml N-acetyl-L-cysteine, 2 μg/ml heparin, penicillin-streptomycin, glutamine 1% (Sigma Aldrich G7513) supplemented with 1% FBS. Whenever appropriate, vitamin A-supplemented B27 (Life Technologies, #17504044) was used.

### EdU incorporation experiments

1 week old S-cNS culture was treated with EdU for 24 h. Spheroids were then embedded in OCT as described below. Incorporated EdU was labeled performing Click-iT Plus reaction according to the manufacturer’s instructions (Life Technologies, Carlsbad, CA, USA, C10632 AlexaFluor 488).

### Protein extraction and western blot

Total protein extracts, SDS-PAGE separation and Western Blot were performed with standard methods as described elsewhere^57,58^. Antibodies were as follows: goat anti-β-actin #SC-1616, mouse anti-α Tubulin TU-02 #SC-8035, rabbit anti-CCND1 #SC-717 and mouse anti-MYCN #SC-53993, mouse anti-Vinculin #SC-73614, mouse anti-Math1 (DSHB), goat anti-DCX # SC-8066 (Santa Cruz Biotechnology); rabbit anti-PARP1 #9542, mouse anti-Gli1 #L42B10, rabbit anti-cleaved caspase-3 #9661 (Cell Signaling Technology Inc); rabbit anti-Myc #C3956 (Sigma Aldrich); rabbit anti-ZIC1 ab72694 (ABCAM), rabbit anti-Pax6 #PRB-278P (BioLegend), mouse anti-Nestin ab11306 (ABCAM). All antibodies were previously used in^56,59–63^. Immunoreactive bands were visualized by enhanced chemoluminscence using WesternBright ECL HRP substrate (Advansta).

### Immunohistochemistry and immunofluorescence assays

Formalin fixed and paraffin embedded tissue sections (4 microns thickness) were processed for H&E staining or probed with Ki67 specific antibody #MA5-14520 (Thermo Fisher Scientific), according to the manufactory instruction of mouse2mouse HRP ready to use kit (MTM001, ScyTek Laboratories, Logan, UT, USA). Images were captured using the microscope Leica DM1000.

For histological analysis, neurospheres were fixed with fresh 4% formalin in 0.1 M of phosphate buffer, pH 7.2, for 24 hours at 4 °C. After washing in PBS, neurospheres were centrifuged at 1200 rpm for three minutes at room temperature and frozen in optimal cutting temperature compound. Three- to four-micron thick cryosections were stained with Haematoxylin and Eosin and scanned via Aperio Scan Scope CS (Leica Byosystem Imaging 0 and analyzed using the Aperio ImageScope™ program (v12.3.2.8013) to measure neurospheres diameter. Alternatively, the cryosections were analyzed by immunofluorescence assay. They were washed with Glycine 1 M for 15’, permeabilized in 0.5% Triton X-100 for 15’and blocked in 5% BSA plus 3% goat serum in PBS. Samples were incubated overnight at 4 °C with anti-DCX # SC-8066, and mouse anti-NESTIN #SC-33677 (Santa Cruz Biotechnology) and revealed with AlexaFluor 488 and 546 secondary antibodies (Life Technologies), respectively.

Adherent cells were fixed in 3,7% formaldehyde/PBS for 15 minutes at RT and processed as described above. Samples were incubated overnight at 4 °C with mouse anti-GFAP #556327 (BD Pharmingen), mouse anti-β3-tubulin #MAB1637 (Millipore), guinea pig anti-VGLUT1 #135304 (Synaptic Systems) and rabbit anti-GABRA6 #AB5610 (Chemicon), and revealed with AlexaFluor 488 and 594 secondary antibodies (Life Technologies).

The acquisition of the images was performed through a Nikon Eclipse Ti equipped with X-Light V2 spinning disk (CrestOptics), LDI laser source (89 North) and Prime BSI Scientific CMOS (sCMOS) camera with 6.5 µm pixels (Photometrics). The images were acquired by using Metamorph software version 7.10.2. (Molecular Devices) with a 100x PlanApo l oil objective (1.4 numerical aperture) and sectioning the slice in Z with a step size of 0.1 um.

Images were deconvolved using the Huygens deconvolution software (Huygens, Hilversum, Netherlands) and analyzed using ImageJ and Photoshop software.

Contrast images of neurospheres were acquired on an EVOS XL CORE 10X microscope. Fluorescence images of Fig. S3 were acquired on a LEICA DM 2500 microscope using the IScapture software.

### RNA extraction and Q-PCR analysis

mRNA was extracted using TRIzol reagent (Invitrogen, Carlsbad, CA, USA), purified with DNase and quantitative reverse transcription-PCR (Q-PCR) was performed as previously described^57,61^ on a ViiA 7 Real-Time PCR System (Thermo Fisher Scientific), using a custom 384-Well Microfluidic Card TaqMan Gene Expression Assay (Thermo Fisher Scientific). The list of the Q-PCR assays is given in Supplementary Table 1. Three biological replicates were analysed for each experimental condition. mRNA expression levels were normalized on the mean of expression of four reference genes: Pgk1, Hprt, Gusb, Tfrc. Heatmaps were generated using the Morpheus analysis (https://software.broadinstitute.org/morpheus).

### Statistical analysis

Data are presented as mean ± SD from three independent experiments (if not otherwise specified). Statistical analysis was performed by a standard two-tailed Student’s *t* test.

## Supplementary information

Supplementary Information

## Data Availability

Data within the manuscript (materials, data and associated protocols) are available from the corresponding author upon reasonable request.
