# Supplementary material for: SMO-M2 mutation does not support cell-autonomous Hedgehog activity in cerebellar granule cell precursors
Source: Sci Rep. 2019 Dec 23;9:19623. doi: 10.1038/s41598-019-56057-y (PMC6928071; doi:10.1038/s41598-019-56057-y)
Supplement: Supplementary file 1 — Supplementary Information [file 41598_2019_56057_MOESM1_ESM.pdf]

**SMO-M2 mutation does not support cell-autonomous Hedgehog activity in cerebellar granule cell precursors**

Marialaura Petroni, Maria Sahùn Roncero, Valentina Ramponi, Francesca Fabretti, Vittoria Nicolis Di Robilant, Marta Moretti, Vincenzo Alfano, Alessandro Corsi, Simone De Panfilis, Maria Giubettini, Stefano Di Giulio, Carlo Capalbo, Francesca Belardinilli, Anna Coppa, Francesca Sardina, Valeria Colicchia, Flaminia Pedretti, Paola Infante, Beatrice Cardinali, Alessandra Tessitore, Gianluca Canettieri, Enrico De Smaele, Giuseppe Giannini

**Supplementary figures, figure legends and tables**

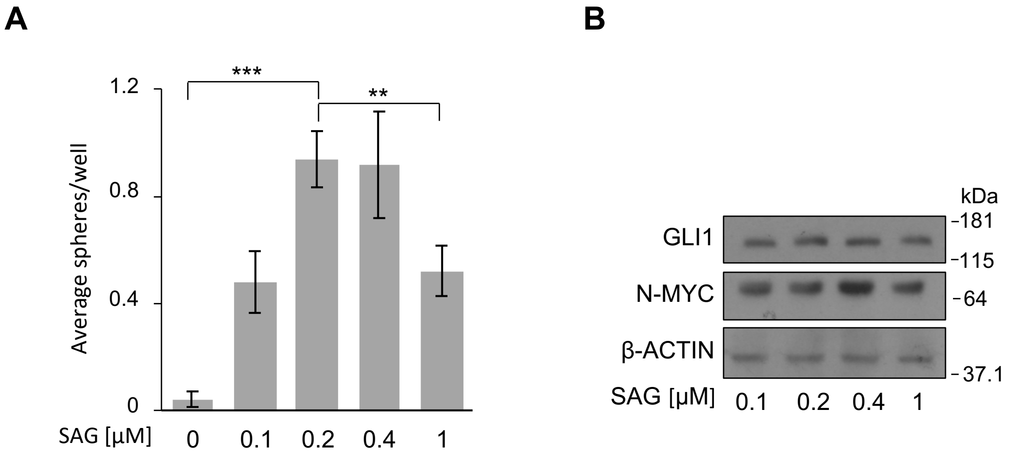


Petroni et al.

**Figure S1**

**Dose-response analysis for SAG-dependent formation of neurosphere and activation of the SHh pathway. A,** P7 WT cerebellar explants were subjected to sphere formation assay with the indicated SAG concentrations. Data obtained by two independent experiments are reported as means +/- SD. (**$p < 0.01$; ***$p < 0.001$). **B,** Protein extracts were obtained from 2 weeks old S-cNS grown at the indicated SAG concentrations and analyzed by WB for the expression of the indicated proteins.

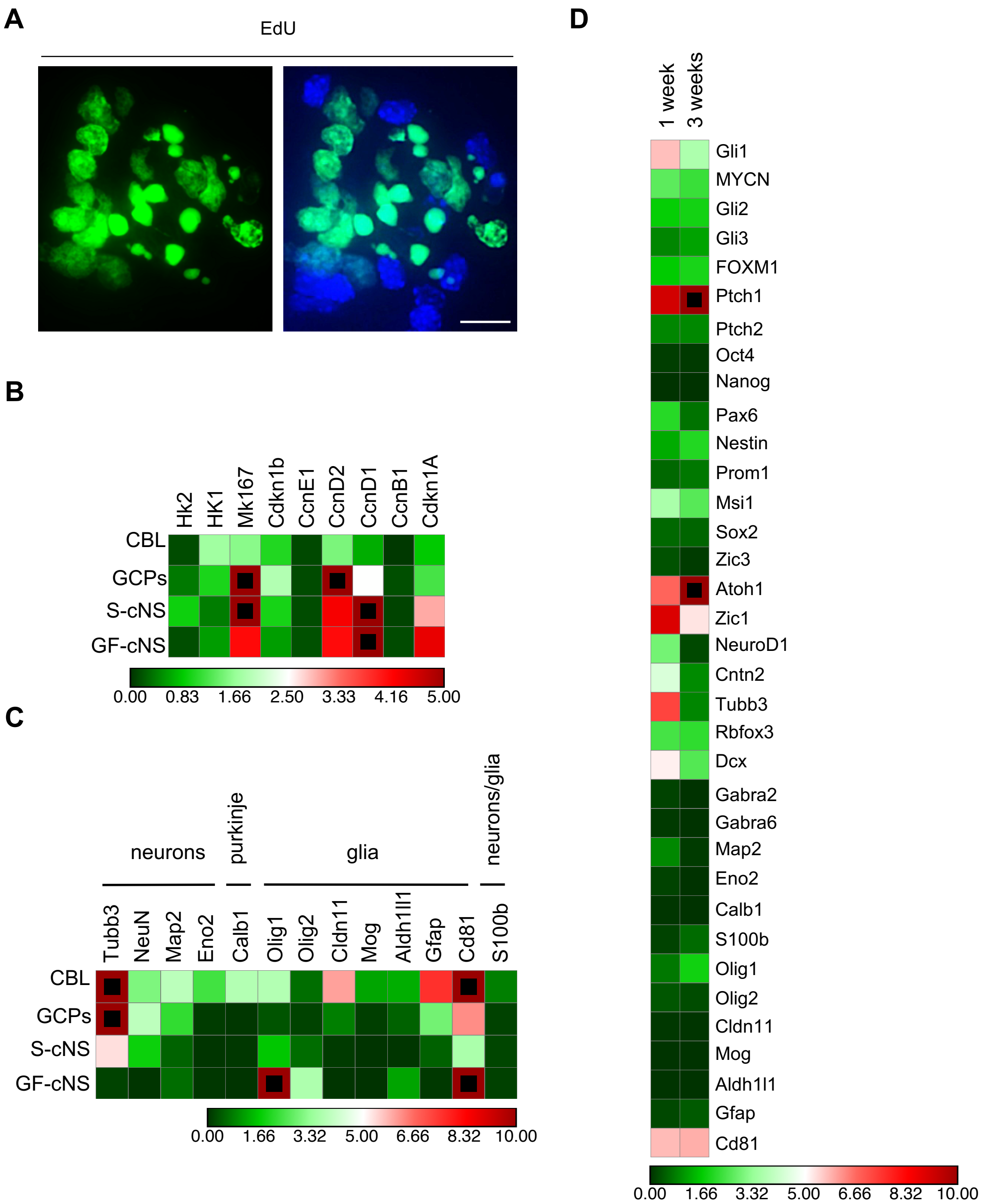


Petroni et al.

**Figure S2**

**Cell proliferation assay and expression profile of selected target genes in S+cNS compared to GF-cNS. A,** EdU incorporation assay was used to measure in S-cNS . S-cNS growing in culture for 1 week were exposed to a single EdU (green) pulse on the last 24 hours. Cell nuclei are evidenced (in blue) by HOECHST- staining. Scale bar, 10 µm. **B**, **C,** Real Time PCR quantification of the indicated transcripts in total cerebellar extracts (CBL), standard GCPs cultures, S-cNS, and GF-cNS. We used three independent samples for each experimental condition. Transcripts expression was normalized on the mean expression level of four reference genes: Pgk1, Hprt, Gusb, Tfrc. Black dots: fold changes exceeding the maximum value of the given scale. **D**, Real Time PCR quantification of the indicated transcripts in 1 and 3 weeks old S-cNS. Normalization was performed as above.

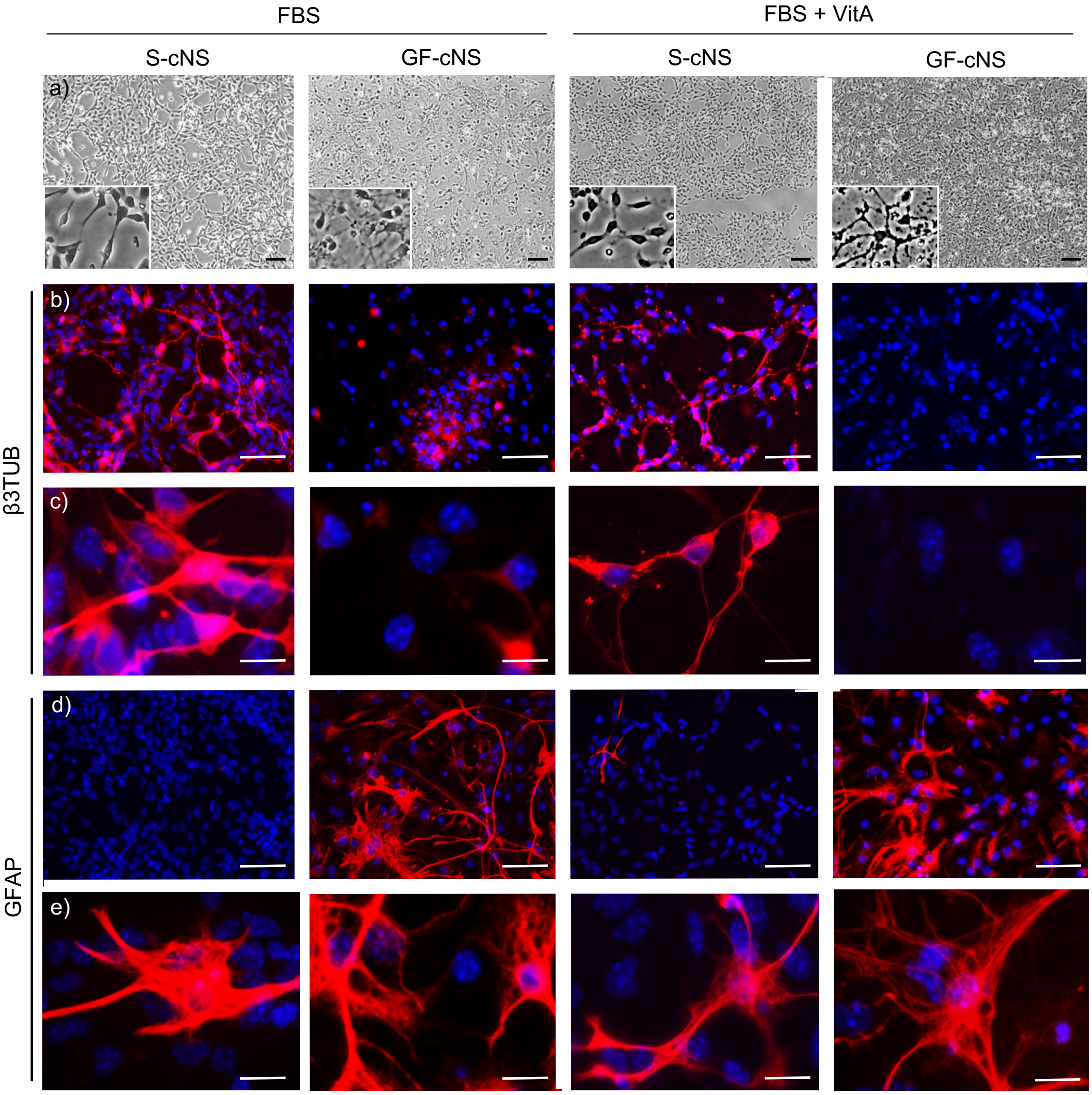


Petroni et al.

**Figure S3**

**S-cNS differentiate into neuron-like cells.** Contrast microphotographs and immunostaining for β-3TUBULIN (red) and GFAP (red) proteins in S-cNS and GF-cNS dissociated and seeded onto polylysine-coated substrates. Cells were treated with differentiation medium containing FBS or FBS+ VitA, for 48 hours. Cell nuclei are evidenced (in blue) by HOECHST- staining. The images are representative of three independent experiments. Scale bar, 50 µm (a, b and d) and 10 µm (c and e).

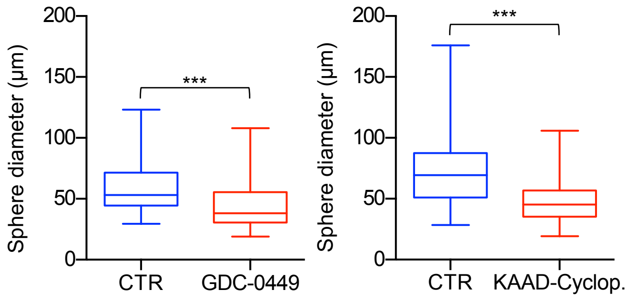


Petroni et al.

**Figure S4**

**Inhibition of the SHh pathways reduces the diameter of S-cNS.**

Box plots representing the diameter of S-cNS treated as in Figure 4C. *$P < 0.05$; ***$P < 0.001$

**A**

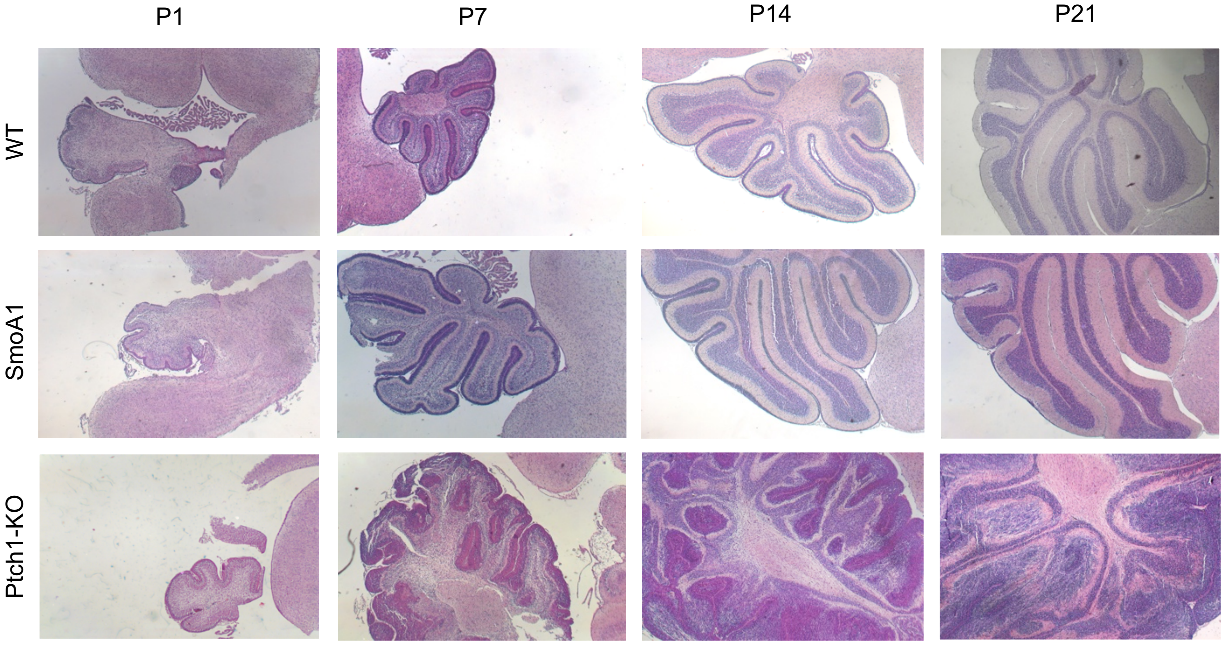


**B**

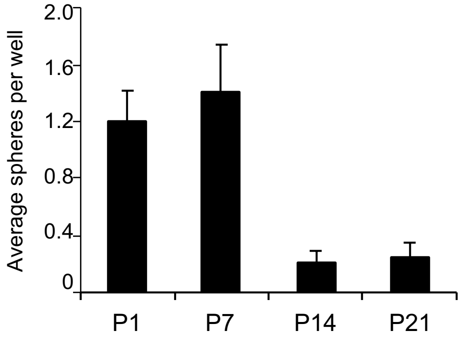


Petroni et al.

**Figure S5**

**Postnatal developmental features of the cerebellum in WT, SmoA1 and Ptch1-KO mouse models.** **A**, Representative sagittal sections of WT, SmoA1 and Ptch1-KO mice from P1 to P21, stained with hematoxylin-eosin (magnification 4x). **B**, Sphere formation assay from WT cerebellar explants performed at the indicated developmental stages.

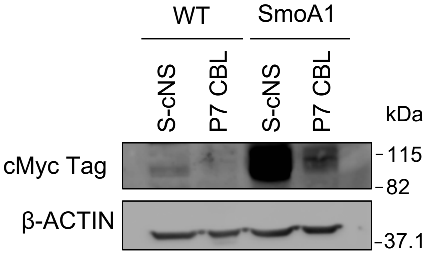


Petroni et al.

**Figure S6**

**SmoA1 transgene expression in S-cNS. A**, Western blot evaluation of SmoA1 transgene expression, revealed by Myc-tag immunostaining, in S-cNS and cerebellar extracts. WT S-cNS and cerebellar extracts were used as negative control for the expression of the transgene. Blot was probed with β-actin as a loading control.

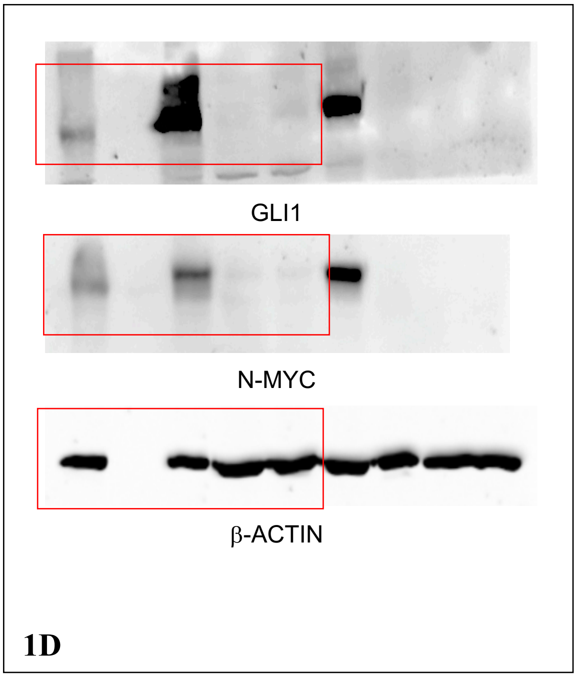


Petroni et al.

**Figure S7**

Uncropped Western blots related to main Fig.1.

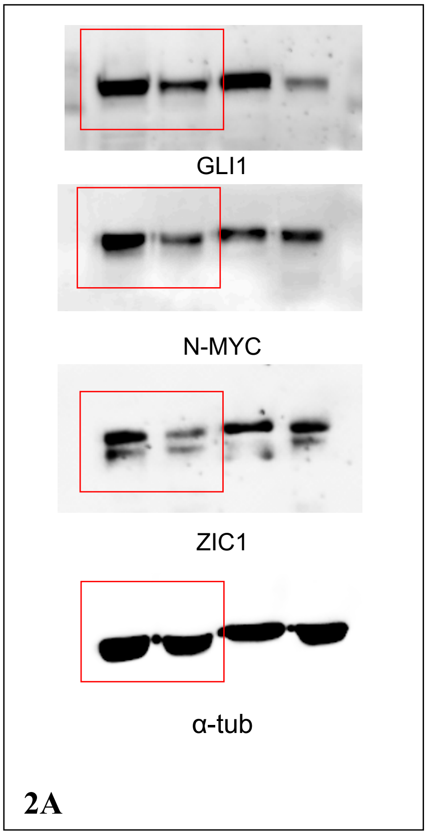


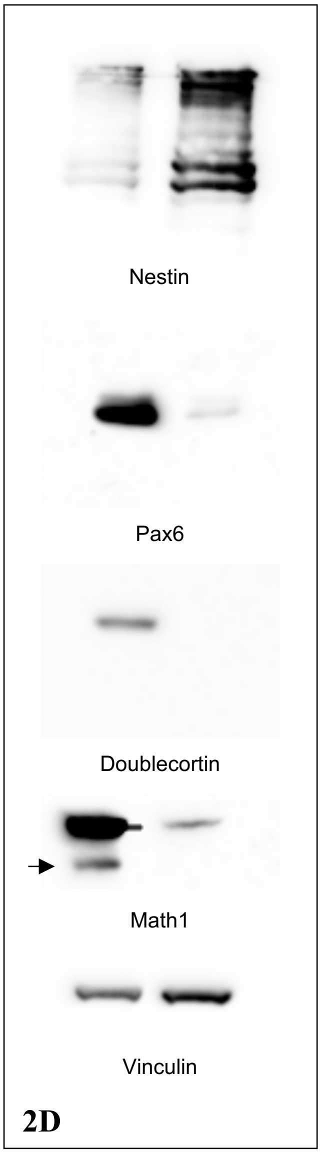


Petroni et al.
**Figure S8**
Uncropped Western blots related to main Fig.2.

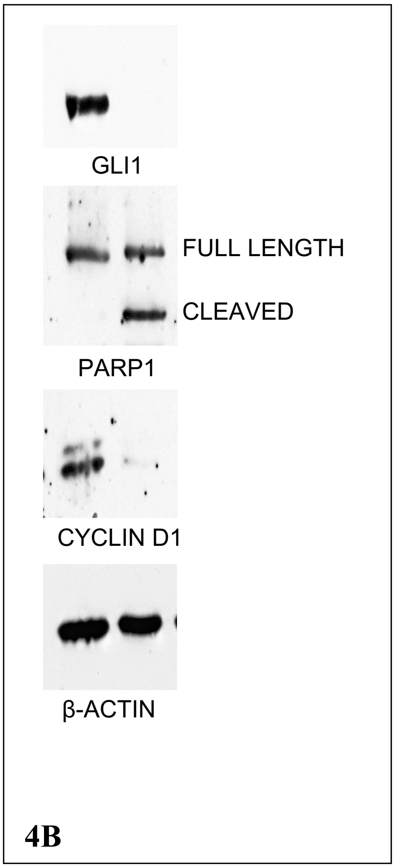


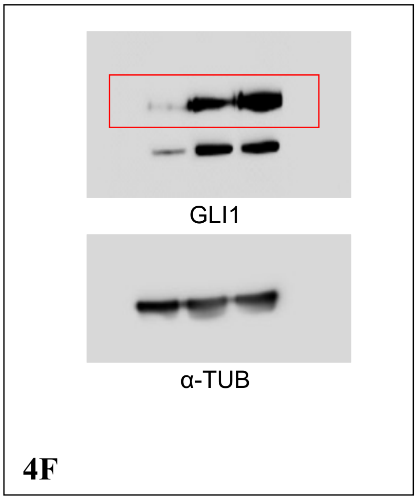


Petroni et al.

**Figure S9**

Uncropped Western blots related to main Fig.4.

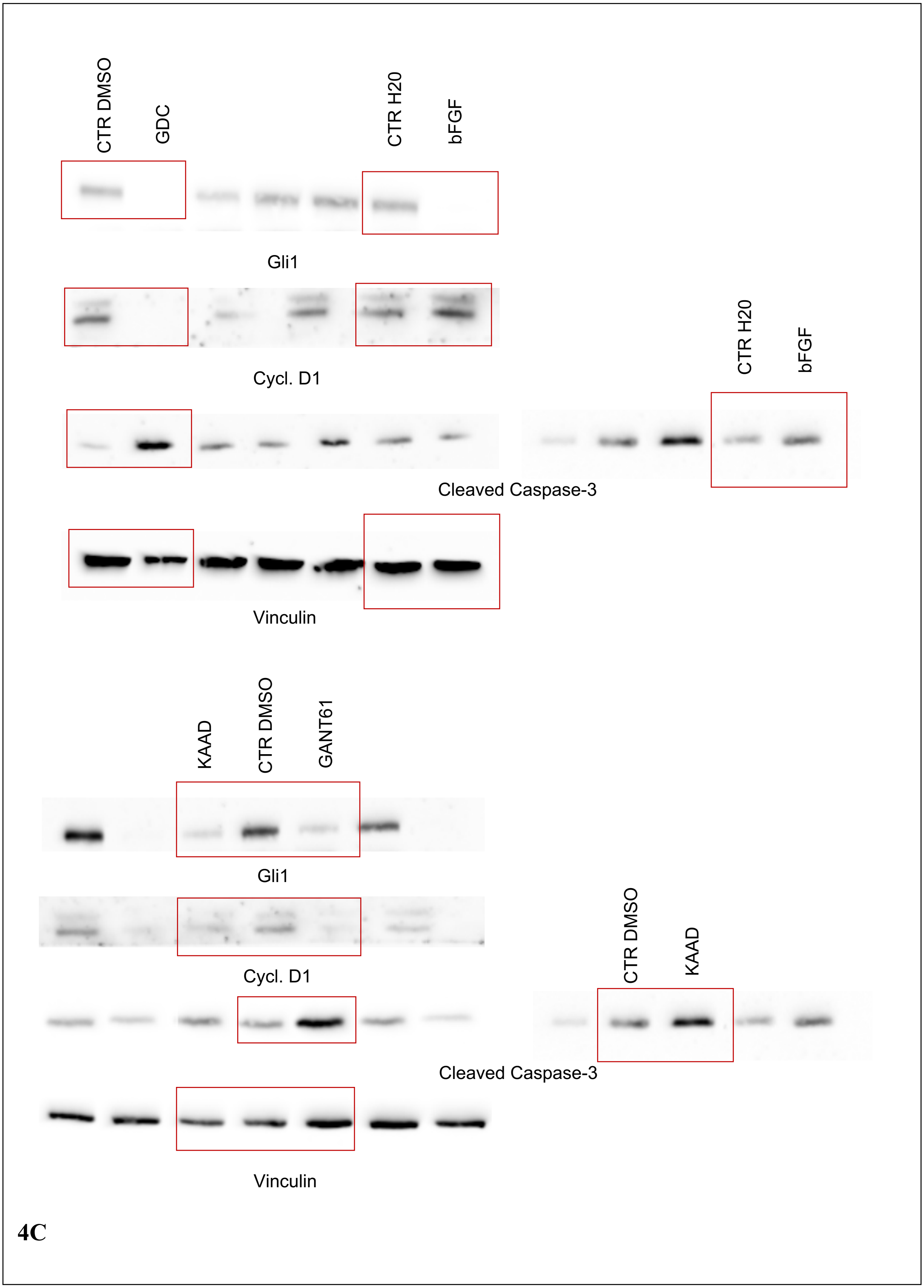


Petroni et al.
**Figure S10**
Uncropped Western blots related to main Fig.4.

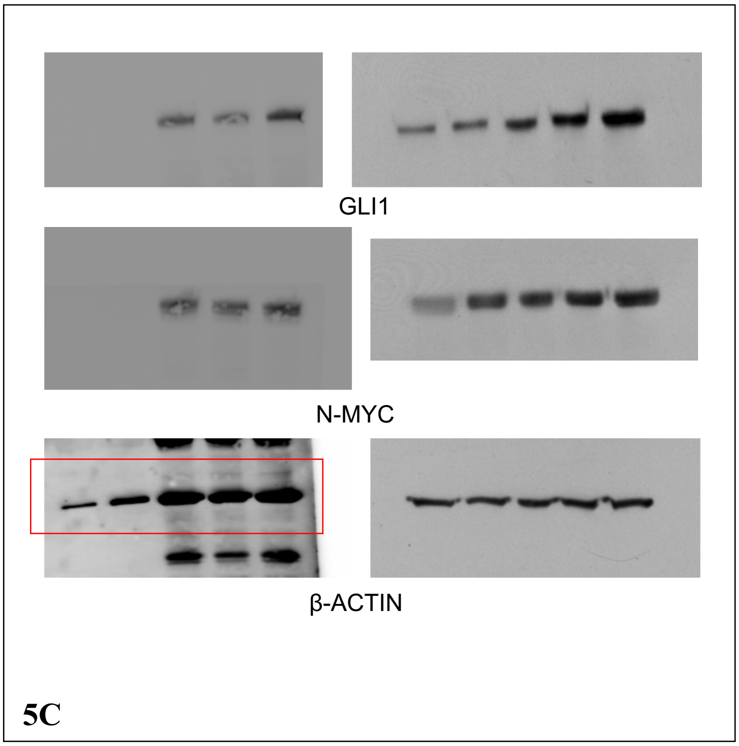


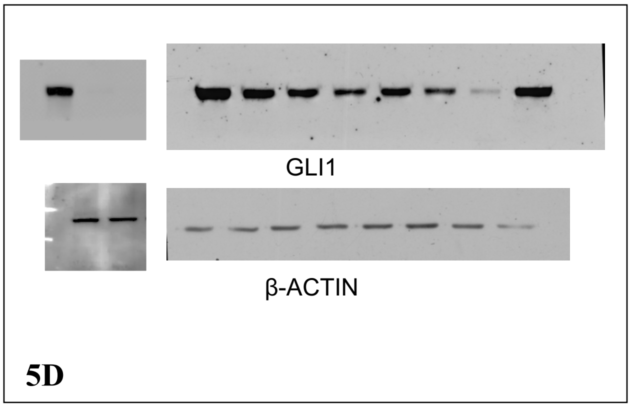


Petroni et al.

**Figure S11**

Uncropped Western blots related to main Fig.5.

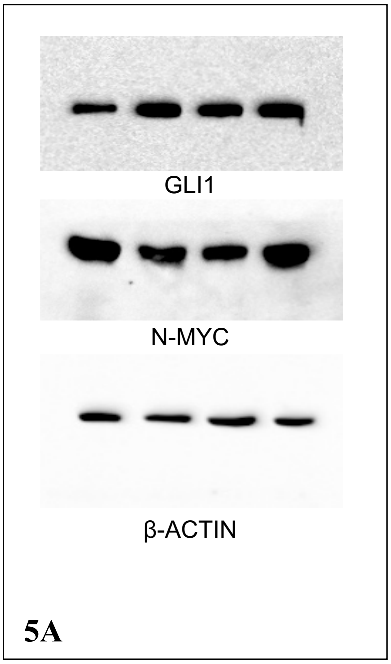


Petroni et al.

**Figure S12**

Uncropped Western blots related to main Fig. 6.

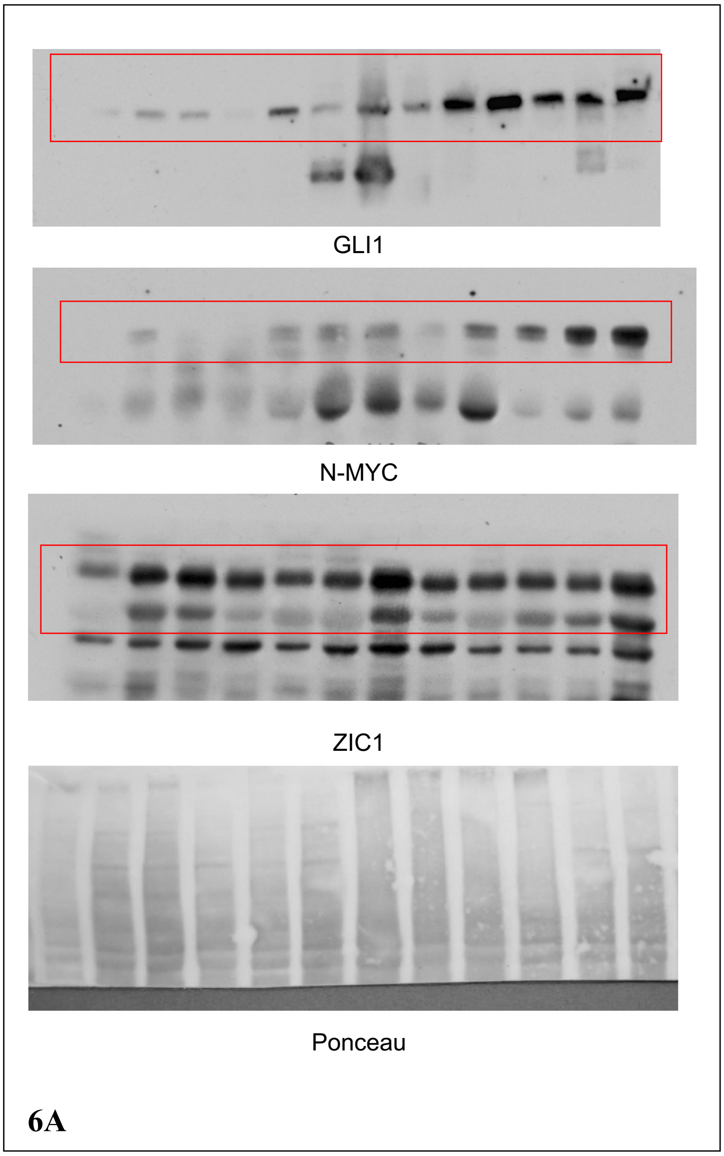


Petroni et al.

**Figure S13**

Uncropped Western blots related to main Fig. 7.

## Table S1

List of assays used for mRNA analysis

| Gene | Assay ID |
|---|---|
| **Aldh1l1** | Mm03048957_m1 |
| **Atoh1** | Mm00476035_s1 |
| **Ccnb1** | Mm02015429_g1 |
| **Ccnd1** | Mm00432359_m1 |
| **Ccnd2** | Mm00438070_m1 |
| **Ccne1** | Mm01266311_m1 |
| **Cd81** | Mm00504870_m1 |
| **Cdkn1a** | Mm00432448_m1 |
| **Cdkn1b** | Mm00438168_m1 |
| **Cldn11** | Mm00500915_m1 |
| **Cntn2** | Mm00516138_m1 |
| **Dcx** | Mm00438400_m1 |
| **Foxm1** | Mm00514924_m1 |
| **Gabra2** | Mm00433435_m1 |
| **Gabra6** | Mm01227754_m1 |
| **Gfap** | Mm01253033_m1 |
| **Gli1** | Mm00494654_m1 |
| **Gli2** | Mm01293117_m1 |
| **Gli3** | Mm00492337_m1 |
| **Gusb** | Mm00446954_g1 |
| **Hprt1** | Mm00446966_m1 |
| **Map2** | Mm00485236_m1 |
| **Mki67** | Mm01278617_m1 |
| **Mog** | Mm00447824_m1 |
| **Msi1** | Mm01203522_m1 |
| **Mycn** | Mm00476449_m1 |
| **Nanog** | Mm01617762_g1 |
| **Nes** | Mm00450205_m1 |
| **Neurod1** | Mm01946604_s1 |
| **Olig1** | Mm00497537_s1 |
| **Olig2** | Mm01210556_m1 |
| **Pax6** | Mm00443081_m1 |
| **pgk1** | Mm00435617_m1 |
| **Pou5f1(OCT4)** | Mm03053917_g1 |
| **Prom1** | Mm00477115_m1 |
| **Ptch1** | Mm00436026_m1 |
| **Ptch2** | Mm01187196_g1 |
| **Rbfox3 (NeuN)** | Mm01248771_m1 |
| **S100b** | Mm00485897_m1 |
| **Sox1** | Mm00486299_s1 |
| **Sox2** | Mm03053810_s1 |
| **Tfrc** | Mm00441941_m1 |
| **Tubb3** | Mm00727586_s1 |
| **Zic1** | Mm00656094_m1 |
| **Zic3** | Mm00494362_m1 |
